# Metagenomic analysis reveals oropharyngeal microbiota alterations in patients with COVID-19

**DOI:** 10.1038/s41392-021-00614-3

**Published:** 2021-05-13

**Authors:** Shengli Ma, Fan Zhang, Fengxia Zhou, Hui Li, Wenyu Ge, Rui Gan, Huan Nie, Biao Li, Yindong Wang, Meng Wu, Duo Li, Dongmei Wang, Zheng Wang, Yuhong You, Zhiwei Huang

**Affiliations:** 1 Heilongjiang Provincial Hospital, Harbin Institute of Technology, Harbin, China; 2 School of Life Science and Technology, Harbin Institute of Technology, Harbin, China; 3 School of Energy Science and Engineering, Harbin Institute of Technology, Harbin, China; 4 Department of Stomatology, Zhongshan Hospital, Fudan University, Shanghai, China

**Keywords:** Microbiology, Biomarkers

## Abstract

COVID-19 remains a serious emerging global health problem, and little is known about the role of oropharynx commensal microbes in infection susceptibility and severity. Here, we present the oropharyngeal microbiota characteristics identified by shotgun metagenomic sequencing analyses of oropharynx swab specimens from 31 COVID-19 patients, 29 influenza B patients, and 28 healthy controls. Our results revealed a distinct oropharyngeal microbiota composition in the COVID-19 patients, characterized by enrichment of opportunistic pathogens such as *Veillonella and Megasphaera* and depletion of *Pseudopropionibacterium, Rothia, and Streptococcus*. Based on the relative abundance of the oropharyngeal microbiome, we built a microbial classifier to distinguish COVID-19 patients from flu patients and healthy controls with an AUC of 0.889, in which *Veillonella* was identified as the most prominent biomarker for COVID-19 group. Several members of the genus *Veillonella*, especially *Veillonella parvula* which was highly enriched in the oropharynx of our COVID-19 patients, were also overrepresented in the BALF of COVID-19 patients, indicating that the oral cavity acts as a natural reservoir for pathogens to induce co-infections in the lungs of COVID-19 patients. We also found the increased ratios of *Klebsiella sp*., *Acinetobacter sp*., and *Serratia sp*. were correlated with both disease severity and elevated systemic inflammation markers (neutrophil–lymphocyte ratio, NLR), suggesting that these oropharynx microbiota alterations may impact COVID-19 severity by influencing the inflammatory response. Moreover, the oropharyngeal microbiome of COVID-19 patients exhibited a significant enrichment in amino acid metabolism and xenobiotic biodegradation and metabolism. In addition, all 26 drug classes of antimicrobial resistance genes were detected in the COVID-19 group, and were significantly enriched in critical cases. In conclusion, we found that oropharyngeal microbiota alterations and functional differences were associated with COVID-19 severity.

## Introduction

The outbreak of coronavirus disease 2019 (COVID-19), caused by severe acute respiratory coronavirus 2 (SARS-CoV-2), has become an ongoing global pandemic.^1^ The disease ranges from mild to critical, and most infected people have mild or moderate disease and eventually recover from COVID-19. However, ~5% of patients develop severe to critical disease. Several risk factors, such as genetics, comorbidities, age, and gender have been reported to influence the relative severity of COVID-19 complications.^2,3^ The main complications of severe COVID-19, such as pneumonia and acute respiratory distress syndrome, are suspected to be caused by bacterial superinfections^4^; moreover, 50% of patients with severe COVID-19 who died presented with a secondary bacterial infection.^3^ Antibiotics play a clearly influential role in the treatment outcome of COVID-19. Bacterial superinfections and required antibiotics illustrate the potential importance of bacteria in COVID-19 complications.

A few current studies have explored the function of the microbiome in the development of COVID-19, suggesting possible relationships between the gut,^5^ pulmonary, nasopharyngeal,^6^ or oral microbiome^7^ and COVID-19. Several bacterial taxa in oral or intestinal microbiomes have been found to be associated with disease severity^8^ and can be used to predict the clinical outcomes of COVID-19.^9^ As the major portal of entry for SARS-CoV-2, the human upper respiratory tract contains an airway microbiome representing its microenvironment and serving as an essential component of the airway epithelial barrier.^10^ The epithelial barrier plays an important role during viral infection. Bacteria of the airway microbiome can directly impact influenza virus infection^11–13^ or act indirectly though the host immune system.^14,15^ During viral infection, the balance of the airway microbiome is disrupted to promote the host innate immune response, and bacterial colonization may be associated with this process.^16,17^ The density and diversity of the airway microbiome vary depending on the position within the airways; the oropharynx bears the highest microbiota density in the upper and lower respiratory tracts, thus, the oropharyngeal microbiome is representative of the airway microbiome.^18^ Importantly, microbial immigration and elimination between the oral cavity and the lungs are constant, making the oral and nasal microorganisms the main sources for the pulmonary microbiome.^19^ Oral health was shown to be associated with multiple respiratory diseases.^20,21^ By analyzing the oropharyngeal microbiome of COVID-19 patients, we can obtain a broad view of the microenvironment balance and functional gene changes. The present study used deep sequencing and metagenomic analysis of the oropharyngeal microbiome in 31 COVID-19 patients (mild, moderate, severe, and critical). We included 29 flu patients and 28 healthy individuals as controls to identify unique characteristics of COVID-19. Comparison of the microbial diversity, relative abundances of the bacteria, and metagene functions of the oropharyngeal microbiome of patients with COVID-19 with that of normal subjects and influenza B patients was used to examine the unique landscape of the oropharyngeal microbiome in COVID-19 and to evaluate the correlations between altered oropharyngeal microbiome, involving the bacterial genera of *Veillonella, Klebsiella*, *Acinetobacter*, *Serratia*, etc., and COVID-19 severity. Interestingly, additional metagene function analysis demonstrated that significant changes in the degradation of amino acids and other small molecules occurred in COVID-19 patients and 26 classes of antimicrobial drug resistance genes were predominantly enriched in the critical cases of COVID-19.

## Results

### Host clinical characteristics associated with COVID-19 severity

The basic and clinical characteristics of the COVID-19 patients are shown in Table 1 and Supplementary Table 1. The median age of the COVID-19 patients was 50 years (range 23–86), with a male:female sex ratio of 1.21. A notable feature of the COVID-19 cohort was that some of the individuals with mild infections did not develop obvious symptoms, although significant viral shedding could be detected by RT-PCR. We further analyzed the biochemical parameters of all the COVID-19 patients, thus ten main indicators related to inflammation in blood tests were investigated. In COVID-19 patients, we found that the levels of C-reactive protein and lactose dehydrogenase were obviously higher in critical patients than in other patients (Supplementary Fig. 1 and Supplementary Table 1). However, the lymphocyte count in critical cases was lowest and showed a gradual decline as the disease progressed. A study of 99 COVID-19 patients revealed that lymphocyte levels decreased in 35% of the patients,^22^ suggesting that dysfunctional cell-mediated immunity may occur in COVID-19 patients.^23^ C-reactive protein is an inflammatory marker produced by the liver, indicating that critical cases had higher levels of inflammation. An elevated level of lactate dehydrogenase in blood tests usually indicates tissue damage, which has multiple potential causes, including infection, reflecting the widespread tissue distribution of the infection. We further investigated the correlations between clinical characteristics and COVID-19 severity (mild, moderate, severe, or critical) in 31 COVID-19 patients. Several features, such as the levels of lactose dehydrogenase and C-reactive protein, the neutrophil–lymphocyte ratio (NLR), the neutrophil count and age, were positively correlated with disease severity (Spearman correlation coefficient *Rho*: 0.44~0.83, *P* < 0.05), whereas the lymphocyte count was negatively correlated (*Rho*: −0.39, *P* < 0.05) (Supplementary Fig. 1).

**Table 1:** Subject characteristics

	COVID-19 (*n* = 31)	Flu (*n* = 29)	Normal (*n* = 28)
Age			
Median (range)-years	50 (23–86)	59 (27–83)	37 (22–87)
≤39—No. (%)	6 (19.35%)	7 (24.14%)	14 (50.00%)
40–49—No. (%)	9 (29.03%)	5 (17.24%)	5 (17.86%)
50–59—No. (%)	9 (29.03%)	3 (10.34%)	2 (7.14%)
60–69—No. (%)	4 (12.90%)	3 (10.34%)	5 (17.86%)
≥70—No. (%)	3 (9.68%)	11 (37.93%)	2 (7.14%)
Sex			
Female (%)	14 (45.16%)	9 (31.03%)	16 (57.14%)
Male (%)	17 (54.84%)	20 (68.97%)	12 (42.86%)
Disease severity			
Mild	2 (6.45%)		
Moderate	17 (54.84%)		
Severe	6 (19.35%)		
Critical	6 (19.35%)		
Blood result median (range)			
Leukocyte count (×10^9^/L, 4.00–10.00)	5.96 (3.09–20.43)		
Lymphocyte count (×10^9^/L, 0.80–4.00)	1.05 (0.35–2.96)		
Platelet count (×10^9^/L,100.00–300.00)	196 (86–456)		
Hemoglobin (g/L, 110–150)	131 (98–164)		
C-reactive protein (mg/L, 0–8.00)	12.75 (4.82–200)		
Alanine aminotransferase (U/L, 0–35)	24 (7–72)		
Lactose dehydrogenase (U/L, 109–245)	225 (108–1190)		
D-dimer (μg/ml, 0.00–0.50)	0.49 (0.01–4.05)		
Neutrophil counts (×10^9^/L, 2.00–7.00)	4 (1.58–19.78)		
AMC (absolute monocyte count, ×10^9^/L, 0.12–1.20)	0.38 (0.11–0.98)		
NLR (ratio of neutrophils to lymphocyte)	3.36 (0.82–46)		
PLR (ratio of platelet to lymphocyte)	200 (59.07–500)		
LMR (ratio of lymphocyte to monocyte)	2.73 (1.26–6.66)		

### Oropharyngeal microbiota profile alterations in COVID-19 patients

**Fig. 1:**
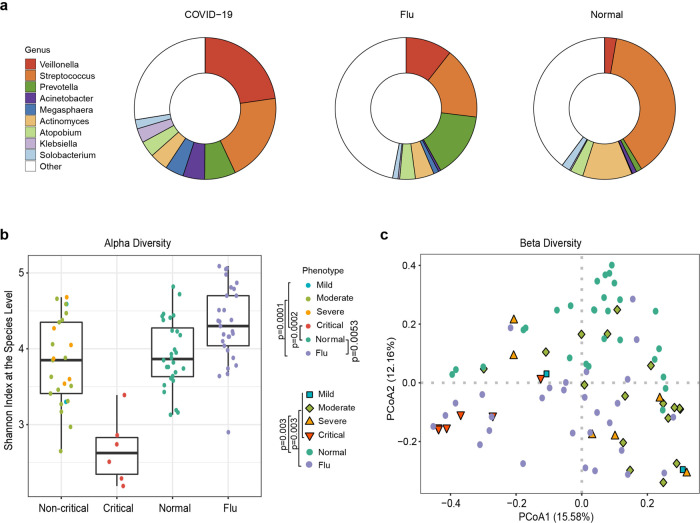
Predominant bacterial composition in COVID-19 patients, flu patients, and healthy controls with α-diversity and PCoA. **a** Fraction of predominant salivary microbiota with a relative abundance of at least 20% at the genus level in COVID-19 patients and their relative proportion in flu patients and healthy controls. **b** α-Diversity in healthy controls (green), flu patients (violet), noncritical COVID-19 patients (mild-blue, moderate-green, severe-orange), and critical COVID-19 patients (red) by the Shannon index at the species level. *N*_mild_ = 2, *N*_moderate_ = 17, *N*_severe_ = 6, and *N*_critical_ = 6. **c** First two axes of PCoA of the Bray distance from the β-diversity of healthy controls, flu patients, and COVID-19 patients. Group differences were tested by pairwise permutational multivariate analysis of variation (PERMANOVA). Mild: blue squares; moderate: green diamonds; severe: orange triangle; critical: red inverted triangle: healthy: green circles; flu: violet circles

To identify alterations in the oropharyngeal microbiome and metagene function changes associated with COVID-19, shotgun metagenomic sequencing data from 88 oropharyngeal swab samples (31 COVID-19, 19 flu, and 28 healthy control samples) that passed quality control were used for metagenomic assembly, microbial annotation and abundance estimation, and metagene functional annotation (Supplementary Table 2). The assembled contigs ranged from 3356 to 842,961 bp in the samples, and the maximum length was 857,671 bp. Finally, a total of 3832,448 nonredundant genes (1943,628 for COVID-19, 1459,770 for flu, and 1556,290 for healthy controls) were obtained (Supplementary Table 3). More than 3000 genera were classified from the oropharyngeal microbiota. The predominant bacterial composition in the COVID-19 group (on average >2% of the total sequences) at the genus level included *Veillonella* (22.7%), *Streptococcus* (20.3%), *Prevotella* (7.1%), *Acinetobacter* (5%), *Megasphaera* (4.21%), *Actinomyces* (4.19%), *Atopobium* (3.65%), *Klebsiella* (3.25%), and *Solobacterium* (2.07%), comprising 75%, 53%, and 60% of the salivary microbiota in the COVID-19 patients, flu patients, and healthy subjects, respectively (Fig. 1a). Next, we compared the microbial diversity of COVID-19 patients with that of flu patients and healthy subjects using the Shannon index for alpha diversity and Bray–Curtis dissimilarities for beta diversity. Alpha diversity describes the species richness within a community, and beta diversity evaluates the species diversity between two communities. The oropharyngeal microbiota in the COVID-19 patients tended to have a lower α-diversity at the species level than that in the flu patients (*P* < 0.01 by the Kruskal–Wallis (KW) test). Among the COVID-19 patients, those who were critical presented a significant diminution in species richness, while the noncritical patients exhibited no significant change from the normal group (Fig. 1b). The above results suggest that there is no significant shift in α-diversity associated with the severity of COVID-19 infection compared with the healthy population; however, the diversity of oropharyngeal microbes was drastically reduced in critical patients. To further test whether the species diversity distinguished the COVID-19 group from the flu and healthy control groups, principal coordinate analysis (PCoA) was applied to compare the β-diversity of the microbial communities, and a marked inter-individual difference was observed between the three groups (*P* < 0.01 by pairwise permutational multivariate analysis of variation) (Fig. 1c), implying that dysbiosis occurred in the COVID-19 oropharyngeal microbiota.

**Fig. 2:**
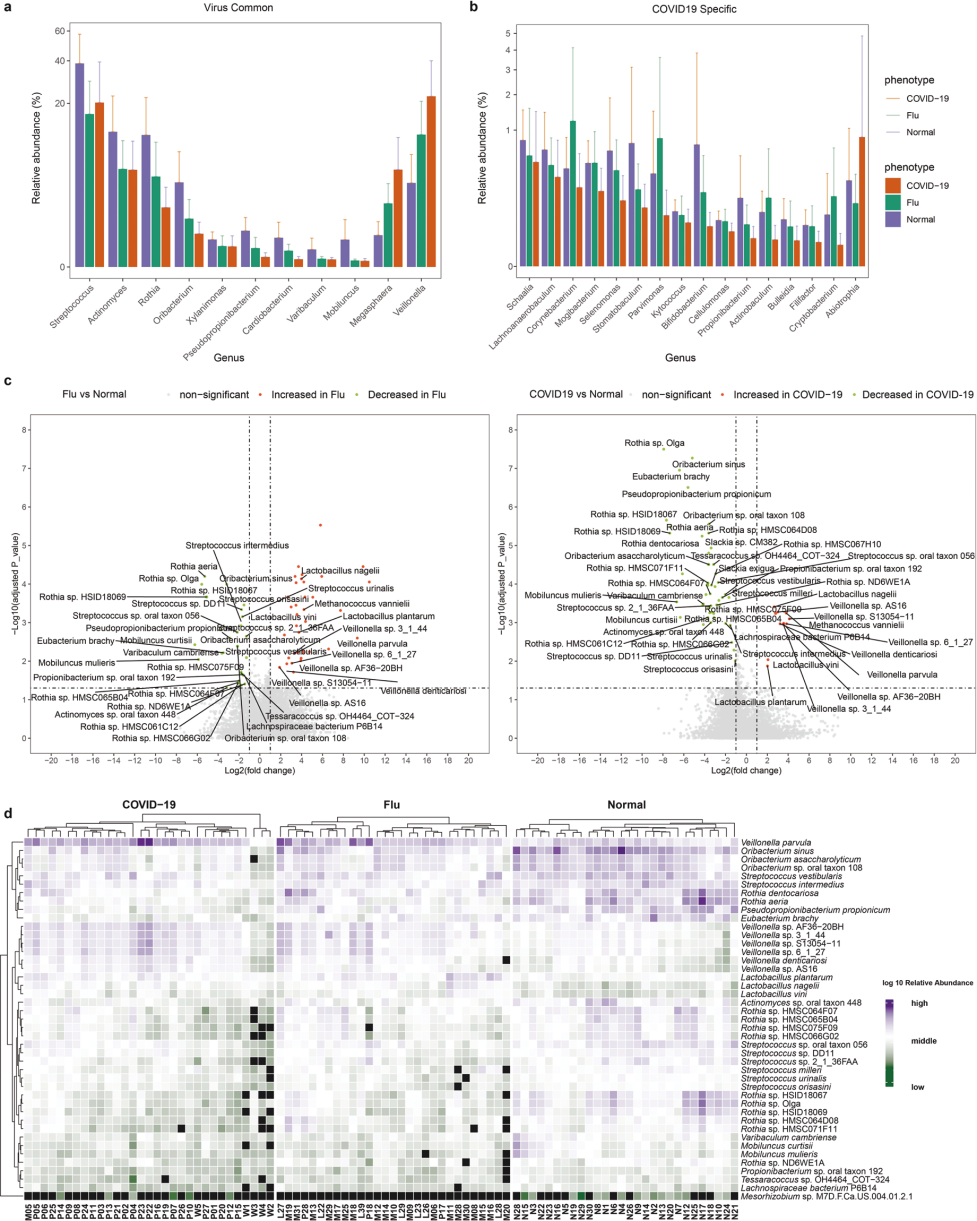
Differentially abundant genera or species in COVID-19 patients, flu patients, and healthy controls. **a** Relative abundance of significantly different genera at the genus level commonly identified by LEfSe when comparing COVID-19 patients with healthy controls or flu patients with healthy subjects. These differentially expressed genera are defined as “virus common.” **b** Relative abundance of significantly different genera at the genus level identified only by LEfSe when comparing COVID-19 patients with healthy controls but not detected when comparing flu patients with healthy subjects. These genera are defined as “COVID-19-specific.” **c** Volcano plot representing the differentially expressed species between flu patients and healthy controls (left), or between COVID-19 patients and healthy controls (right) with the *x*-axis denoting log2(fold-change) and the *y*-axis denoting −log10(adjusted *P* value). The significantly increased species in flu or COVID-19 patients are shown in red. The significantly decreased species in flu or COVID-19 patients are shown in green. Dashed vertical and horizontal lines reflect the filtering criteria (absolute fold-change (FC) ≥ 2.0 and FDR-adjusted *P* value < 0.05). **d** Heatmap depicting the relative abundance of significantly varied species when comparing COVID-19 patients with healthy controls and flu patients with healthy controls. Rows (microbial taxa at the species level) and columns (samples) are ordered by hierarchical clustering based on Euclidean distance. Colors denote log10 relative abundance of each species in each sample. The relative abundance is shown in purple (high), white (middle), green (low), and black (zero)

We next identified the differences in taxa at the genus level by comparing the relative abundance of the microbiota composition of COVID-19 patients with those of flu patients and healthy controls using the linear discriminant analysis effect size (LEfSe) method. We found significantly higher levels of *Veillonella* and *Megasphaera*, and lower levels of *Mobiluncus*, *Varibaculum*, *Cardiobacterium*, *Pseudopropionibacterium*, *Xylanimonas*, *Rothia*, *Oribacterium*, *Actinomyces*, and *Streptococcus*, in both the COVID-19 and flu patients than in the healthy control group, indicating common characteristics correlated with acute respiratory viral infection (Fig. 2a). The relative abundance of *Abiotrophia* was significantly higher, while those of *Cryptobacterium*, *Filifactor*, *Bulleidia*, *Actinobaculum*, *Propionibacterium*, and ten other genera were significantly lower, in the COVID-19 group but not in the flu group compared with the healthy controls, indicating a unique feature associated with COVID-19 (Fig. 2b). Interestingly, *Veillonella* was the most prominent biomarker for the COVID-19 group compared with either the flu patients or healthy controls, and thus, the COVID-19 patients might be designated by the *Veillonella*-dominant cluster.

Furthermore, differential expression analysis of bacterial species between COVID-19 or flu patients and healthy controls was performed by the Wilcoxon rank sum test (with adjusted *P* < 0.05) and among the three groups with the KW test (with adjusted KW *P* < 0.001). In total, 61 and 70 differentiating bacterial species were identified for the COVID-19 and flu patients, respectively, compared with the healthy subjects (Fig. 2c and Supplementary Table 4). Finally, 44 common species that were significantly increased or decreased in both the COVID-19 and flu patients compared with the healthy controls were detected, and their relative abundance is shown in the heatmap (Fig. 2d). These species were clustered into four branches, and belonged predominantly to the *Actinobacteria* and *Firmicutes* phyla. *Veillonella parvula* itself formed a cluster, which occurred in all the samples, and the lowest relative abundance was in the healthy controls, followed by the flu patients, whereas the highest was in the COVID-19 patients. The relative abundance of *Veillonella parvula* in the flu or COVID-19 patients was 9 or 13 times higher than that in the healthy controls (Fig. 2c). Eight species, including *Oribacterium sinus* and *Pseudopropionibacterium propionicum*, formed cluster 2, which was enriched in the healthy controls and relatively depleted in the COVID-19 patients. *Veillonella* sp. AF36-20BH, *Lactobacillus vini*, and nine other species formed cluster 3. The relative abundances of the cluster 3 members were higher in the flu or COVID-19 patients than those in healthy controls, without consistent enrichment or deletion in one group of patients. The remaining 25 species, belonging predominantly to the *Actinomycetales*, *Micrococcales*, and *Lactobacillales* orders, formed a large cluster. These bacteria were enriched in the healthy controls and were reduced in the flu or COVID-19 patients. In summary, our results revealed a distinct oropharyngeal microbiota composition in COVID-19 patients compared to the flu patients and healthy controls, characterized by enrichment of opportunistic pathogens such as *Veillonella* and *Megasphaera* and depletion of *Pseudopropionibacterium, Rothia*, and *Streptococcus*. These results suggest that respiratory viral infections may be associated with an altered oropharyngeal microbiome that predisposes patients to secondary bacterial infections. A previous study revealed that *Veillonella parvula*, *Prevotella melaninogenica*, *Capnocytophaga gingivalis*, and *Leptotrichia buccalis* were overrepresented in the BALF of a COVID-19 patient.^7,24^ These oral opportunistic pathogens were also more abundant in our COVID-19 patient cohort with a 2.2–14-fold increase compared with that in the healthy subjects, indicating that the oral cavity is likely to be a natural reservoir for pathogens inducing co-infections in the lungs of COVID-19 patients (Supplementary Table 4).

### Oropharyngeal microbiota markers correlated with COVID-19 severity

**Fig. 3:**
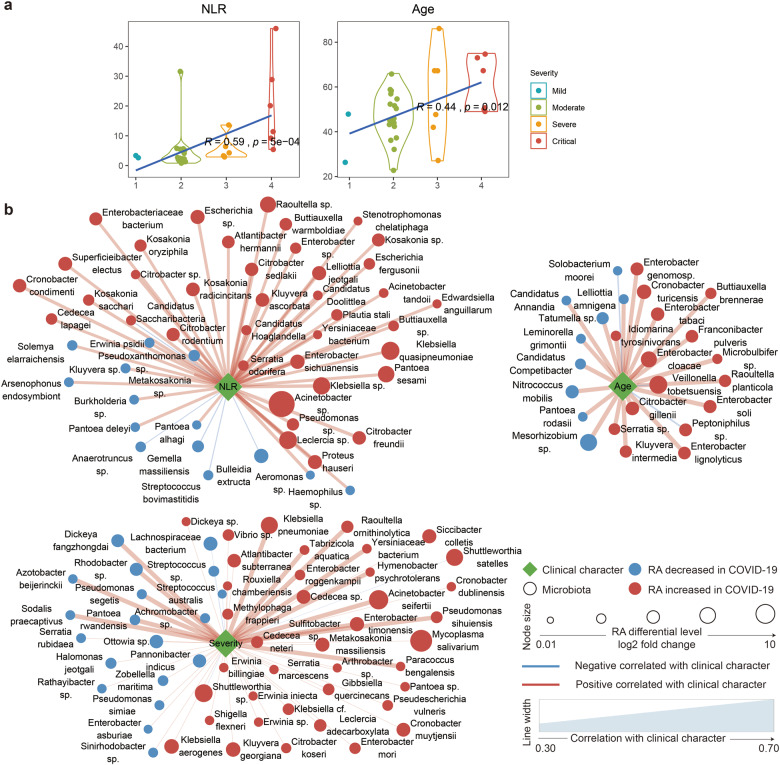
Association between clinical characteristics and microbiota in COVID-19 patients. **a** Violin plot showing the correlation between clinical characteristics (neutrophil–lymphocyte ratio (NLR) and age) and the severity of COVID-19. Spearman correlation was used for correlation analysis. **b** Correlation networks between three clinical characteristics (COVID-19 severity, neutrophil–lymphocyte ratio (NLR), and age) and microbiota at the species level in COVID-19 patients. The nodes represent unique species, and the color of the nodes denotes an increase (red) or decrease (blue) in relative abundance when comparing COVID-19 patients with healthy controls. The size of the nodes shows the log2-fold-change values of the relative abundance in COVID-19 patients versus healthy controls. The edges denote the correlation between the species and the clinical characteristics, the color of the edge represents a positive (red) or negative (blue) correlation, and the width denotes the strength of the correlation

Notably, COVID-19 severity was positively correlated with the NLR (*Rho* = 0.59, *P* = 5e − 04) and age (*Rho* = 0.44, *P* = 0.012) (Fig. 3a). This was also seen by Liu et al.,^25^ who deduced that the NLR could predict the severity of a patient’s response to COVID-19 infection. Then, we examined the association between microbiota, the inflammation-related marker NLR, age and disease severity. In total, 123 and 13 species were positively or negatively correlated with COVID-19 disease severity, respectively (*P* value < 0.001, *Rho* > 0.6 or *Rho* < −0.6, Supplementary Table 5), most of which were also significantly increased (82/123 = 66.67%) or decreased (8/13 = 61.54%) in the COVID-19 patients compared with the healthy subjects (Fig. 3b). Among these species, 59 were also significantly correlated with the NLR. *Klebsiella sp*. (*Rho* > 0.74, *P* < 6.2E − 07), *Acinetobacter sp*. (*Rho* > 0.72, 3.72E − 06), and *Serratia sp*. (*Rho* > 0.72, *P* < 4.8E − 06) were among the species most positively correlated with COVID-19 severity and were also correlated with the systemic inflammatory marker NLR (Rho: 0.35–0.696, *P* < 0.05). These species were found to be indicators of ventilator-associated pneumonia (VAP) in a previous study.^26^ *Veillonella tobetsuensis*, which has been identified as one of the top-three bacterial species for the prediction of COVID-19 severity, was also verified in our COVID-19 cohort (*Rho* < −0.6, *P* < 0.00034). *Streptococcus sp*. and *Peptoniphilus sp*. were the two species most negatively associated with COVID-19 disease severity (*Rho* < −0.67, *P* < 3E − 05). *Peptoniphilus sp*. was abundant in biofilms cultured from different oral niches and its inverse correlation with COVID-19 severity might suggest its protective role in the oral cavity. In summary, these results indicate that salivary microbiota alterations in combination with host systemic inflammatory status, age or immune response may impact disease severity.

### Identification of a microbial classifier for COVID-19

**Fig. 4:**
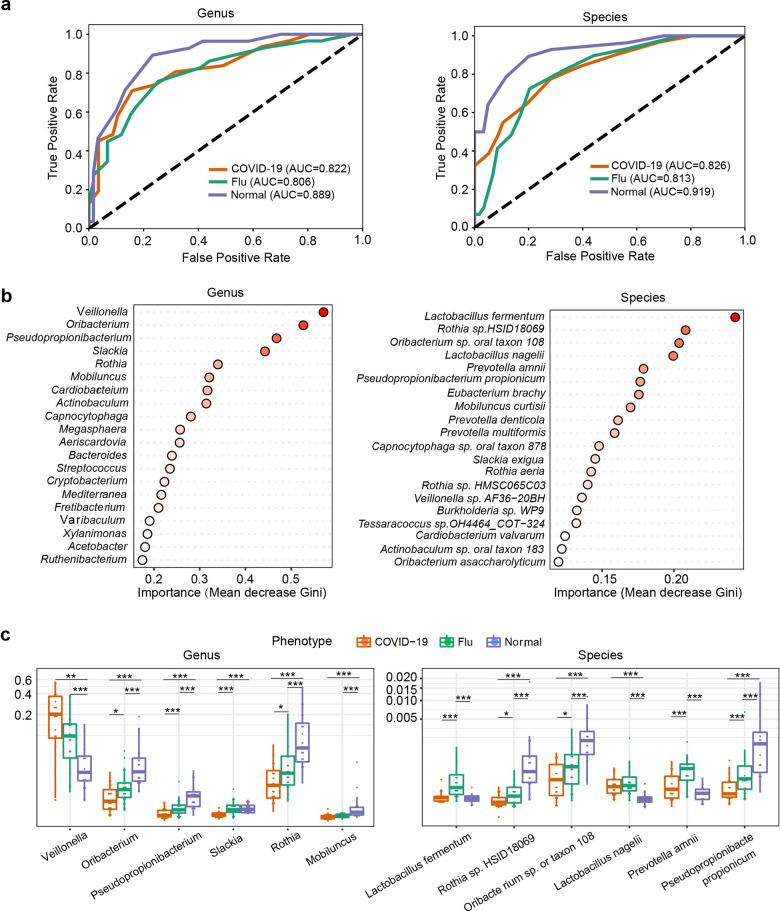
Main bacterial genera and species classified in COVID-19 patients, flu patients, and healthy controls based on random forest analysis. **a** ROC curves showing the discriminative ability among the three groups (COVID-19, flu, and normal) using the relative abundance of the oropharyngeal microbiome at the genus and species levels. **b** Top 20 important genera and species based on Gini importance according to the trained random forest models. **c** Comparison of the relative abundance of the top 6 genera or species selected based on Gini importance by boxplot. ***P < 0.001; **P < 0.01; *P < 0.05 using the Kruskal–Wallis (KW) test

To identify characteristic species associated with COVID-19, an unsupervised random forest classification analysis using a leave-one-out cross-validation procedure was performed. We identified a microbial genus or species classifier distinguishing COVID-19 patients from flu patients and healthy controls with an area under the receiver operating characteristic curve (AUC) of 0.822 or 0.826, and distinguishing patients (combining COVID-19 and flu patients) from healthy controls with an AUC of 0.889 or 0.919 (Fig. 4a). The model identified the top 20 bacterial genera and species among COVID-19 patients, flu patients, and healthy controls (Fig. 4b). The main taxa were the phyla *Firmicutes* and *Actinobacteria*. The relative abundances of the top six genera and species in the three groups are shown in Fig. 4c. *Veillonella* and *Lactobacillus nagelii* were significantly increased in the COVID-19 group *(P* < *0.01*, KW test*)*, compared with healthy controls. Decreased *Mobiluncus* and *Pseudopropionibacterium*, *Oribacterium* sp. oral taxon 108 and *Pseudopropionibacterium propionicum* were found in the COVID-19 patients compared with the healthy controls (*P* < 0.01, KW test) (Fig. 4c).

### Functional potentials of the oropharyngeal microbiome associated with COVID-19

**Fig. 5:**
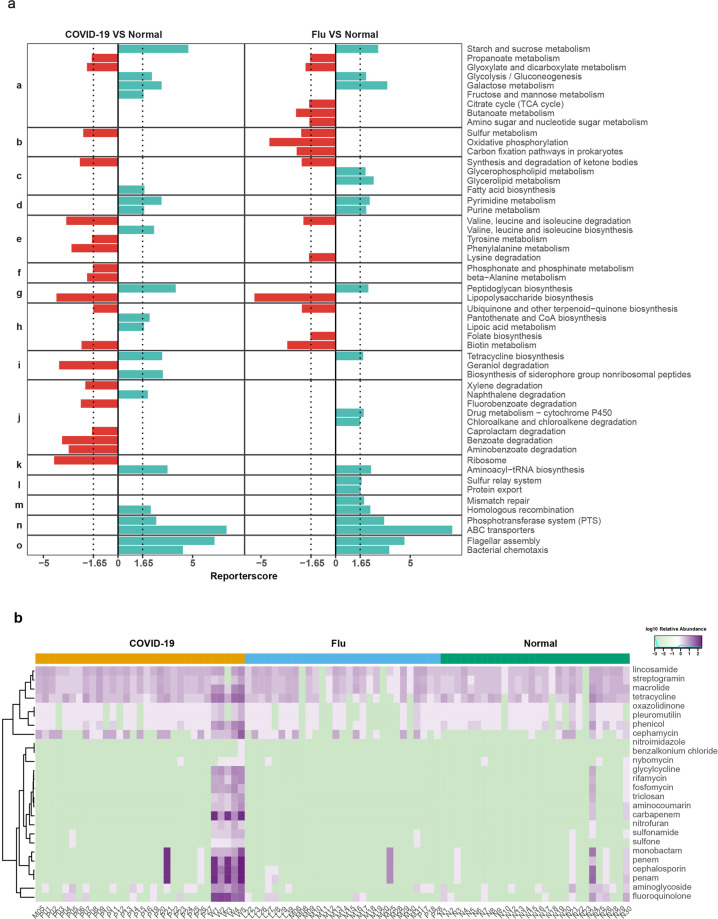
Differential enrichment of KEGG functions in COVID-19 and flu patients. **a** Differential enrichment of KEGG functions in COVID-19 and flu patients: (a) carbohydrate metabolism. (b) Energy metabolism. (c) Lipid metabolism. (d) Nucleotide metabolism. (e) Amino acid metabolism. (f) Metabolism of other amino acids. (g) Glycan biosynthesis and metabolism. (h) Metabolism of cofactors and vitamins. (i) Metabolism of terpenoids and polyketides. (j) Xenobiotic biodegradation and metabolism. (k) Translation. (l) Folding, sorting, and degradation. (m) Replication and repair. (n) Membrane transport. (o) Cell motility. Red, case-enriched and cyan, control-enriched within each disease cohort (NCOVID-19 = 31, NFlu = 29, and NNormal = 28). Dashed lines indicate a reporter score of 1.96, corresponding to the 95% confidence of a normal distribution. **b** Heatmap indicating the relative abundance of AMR genes in 26 drug classes of the three groups

To gain insight into functional changes within the COVID-19 oropharyngeal microbiome, we studied the Kyoto Encyclopedia of Genes and Genomes (KEGG) pathway genes enriched in the oropharyngeal microbiota of the patients compared to the controls (Fig. 5a). The samples from the COVID-19 patients displayed a higher potential for the metabolism of amino acids (valine, leucine, isoleucine, tyrosine, and phenylalanine) and other amino acids (beta-alanine, phosphonate, and phosphinate). Except for valine, leucine, and isoleucine, the abovementioned amino acid metabolism pathways were enriched only in the COVID-19 patients compared to the flu patients and healthy controls. These data indicate that the oropharyngeal microbiome of COVID-19 patients preferentially metabolizes specific amino acids.

Moreover, compared to those of the control and flu samples the oropharyngeal microbiome of the COVID-19 patients exhibited significant enrichment in xenobiotic biodegradation and metabolism. The potential for the degradation of benzoate, fluorobenzoate, aminobenzoate, xylene, and caprolactam was elevated, whereas the potential for the degradation of naphthalene was reduced. For the flu patients, the potential for energy metabolism (carbon fixation pathways in prokaryotes and oxidative phosphorylation) was higher than that in the COVID-19 patients, while sulfur metabolism was similar to that in the COVID-19 patients. Nucleotide metabolism (purine and pyrimidine), replication and repair (homologous recombination and mismatch repair) and folding, sorting and degradation (sulfur relay system and protein export) in the oropharyngeal microbiome of the COVID-19 or flu patients were relatively depleted; however, the other functions in genetic information processing were neither enriched nor depleted. This finding suggests that the oropharyngeal microbiome of the COVID-19 or flu patients may have a lower genetic information processing ability. In particular, membrane transport (ABC transporters and the phosphotransferase system) and cell motility (bacterial chemotaxis and flagellar assembly) were strongly depleted in the oropharyngeal microbiomes of the COVID-19 and flu patients. These data indicate that microorganisms in the throats of COVID-19 and flu patient have less potential for membrane transport of ions, lipids, sterols, peptides, proteins, and carbohydrates, particularly hexoses, hexitols, and disaccharides, with worse cell motility.

Among the oropharynx shotgun metagenomes of the COVID-19, flu, and normal groups, 4639, 473, and 665 antimicrobial resistance (AMR) genes consisting of 26 drug classes (aminocoumarin, aminoglycoside, benzalkonium chloride, carbapenem, cephalosporin, cephamycin, fluoroquinolone, fosfomycin, glycylcycline, lincosamide, macrolide, monobactam, nitrofuran, nitroimidazole, nybomycin, oxazolidinone, penam, penem, phenicol, pleuromutilin, rifamycin, streptogramin, sulfonamide, sulfone, tetracycline, and triclosan) were detected, respectively. All 26 AMR gene drug classes were detected in the COVID-19 patients. In the flu patients and healthy controls, only 17 and 24 AMR gene drug classes were detected. The relative abundances of these 26 drug classes of AMR genes are visualized in the heatmap (Fig. 5b). All groups included common antibiotics such as lincosamide, streptogramin, macrolide, and tetracycline. Benzalkonium and nitroimidazole AMR genes occurred only in the COVID-19 group. The most abundant drug class was penam (274 AMR genes), followed by cephalosporin (261 AMR genes) and penem (256 AMR genes) only in the COVID-19 group. In the flu group, the top four drug classes were macrolide, penam, penem, and cephalosporin. Notably, these four AMR gene drug classes were found in a single flu patient (M25); if we excluded the outlier, the most abundant drug classes were lincosamide and macrolide. In the normal group, the most abundant drug class was penam (33 AMR genes), followed by cephalosporin (32 AMR genes) and cephalosporin (25 AMR genes) in one individual (N24). Similarly, after excluding the outlier, tetracycline was the top AMR gene drug class in the healthy control group. We found that AMR genes were significantly enriched in six COVID-19 patients. Except for patient P21, the five other patients were critical. Patient P21 had moderate COVID-19; however, she experienced several complications (angina pectoris, cholecystitis, gastritis, hypertension, etc.) before COVID-19 (Supplementary Table 1). Complications and poor physical condition may allow more AMR bacteria to colonize the oral cavity or airway.

Several mechanisms of antibiotic resistance were identified in the metagenomic dataset, such as antibiotic target protection proteins, efflux pump complexes or subunits conferring antibiotic resistance, antibiotic target-modifying enzymes, antibiotic inactivation enzymes, etc. The most common resistance mechanism found in this study was the antibiotic inactivation enzyme, a determinant of beta-lactam resistance mediated by *Klebsiella pneumoniae* and *Klebsiella oxytoca*. Xenobiotic biodegradation and metabolism and multiple drug AMR genes were enriched in the COVID-19 patients, which was related to the medical history and medications.

## Discussion

Our study is among the pioneering studies to explore the metagenomic characteristics of the oropharyngeal microbiome in COVID-19 patients with various severity (mild, moderate, severe, or critical) compared with flu patients and healthy controls, since we started to collect the samples on Jan 20^th^, 2020. Bioinformatics analysis of the metagenomic sequencing data obtained in the present study showed that SARS-CoV-2 infection altered the composition of the oropharyngeal microbiota and caused dysbiosis of the local microbiome, which may induce translocation of oral pathogens into the lungs to cause pulmonary co-infections.

Similar to a dramatic decrease in the oral microbiome diversity due to predominance of a certain microbiome in a severe infection reported previously,^27,28^ we found that the diversity of oropharyngeal was decreased in COVID-19 patients, reaching a greater significance in critical patients (Fig. 1) and indicating the presence of dysbiosis in the oropharyngeal microbiota of COVID-19. Hence, we hypothesized that the genera of oral bacteria enriched in COVID-19 such as *Veillonella and Megasphaera* may be pathogenic when transferred to other organs of the body. Convincing data indicated that the levels of eight out of ten species overrepresented in the BALF of COVID-19 patients^24^ were increased in the COVID-19 oropharyngeal microbiome in the present study, confirming that the oral cavity may be a source of the pathogens that infect the lung. A number of studies have demonstrated a link between good oral care and a reduced risk of respiratory tract infection^29,30^ and pneumonia-related mortality in elderly people.^31,32^ Thus, the results of the present study are clinically significant because variations in the oropharyngeal microbiome of COVID-19 patients can be used as noninvasive biomarkers of dysbiosis of the pulmonary microbiome or of invasion of potential pathogens in the lung.

The *Veillonella* genus has been shown to be a shared indicator of COVID-19 in multiple studies,^9,33^ a cause of chronic anaerobic pneumonitis,^34^ and present at a high abundance in the oral cavity of individuals with rheumatoid arthritis.^27^ Several members of the *Veillonella* genus are periodontal pathogens and are overrepresented in the BALF of COVID-19 patients.^7,24^ In addition, we generated a microbial classifier to distinguish COVID-19 patients from flu patients and healthy controls with an AUC of 0.889, and the classifier identified *Veillonella* as the top predictor. Thus, *Veillonella* may contribute to the severity of COVID-19, although the exact mechanism of the effect requires further exploration.

Many risk factors have been reported to influence COVID-19 severity,^3^ including sex, age, and comorbidities. However, a substantial proportion of apparently healthy infected patients with no identified risk factors also suffer from severe complications, suggesting that other risk factors, such as oral hygiene or microbiome dysbiosis, should be considered. This hypothesis is supported by the data of the present study showing an association between the clinical characteristics of the host and COVID-19 severity. Age and several indicators linked to the inflammatory response in blood tests, including NLR, were significantly correlated with disease severity (Spearman correlation analysis, *P* < 0.05 Supplementary Fig. 1), indicating that the inflammatory response may play a critical role in the development of severe disease forms. Both the NLR and age were correlated with COVID-19 severity (Fig. 3a); however, the NLR was not related to age (*Rho* = 0.21, *P* = 0.26). Therefore, the NLR and age may be distinct factors contributing to COVID-19 severity. A higher NLR value has been shown to indicate a higher probability of bacterial infection and a lower probability of viral infection,^35^ implying that bacterial superinfections may supersede the original viral infection in severe cases of COVID-19. Moreover, microbiome dysbiosis can promote an inflammatory environment favoring coronavirus invasion and viral replication,^5^ thus contributing as a risk factor for disease severity. In the present study, 136 species positively or negatively correlated with COVID-19 severity included 53 (39%) and 25 (18.4%) species, which were also correlated with host inflammatory NLR status and age, respectively. These results indicated that alterations in the oropharyngeal microbiota may impact disease severity due to interactions with the systemic inflammatory status of the host, age, or immune response. Several species with the highest correlations with COVID-19 severity in the present study, such as *Klebsiella sp*., *Acinetobacter sp*., *Serratia sp*., and *Veillonella tobetsuensis*, were also identified as indicators for VAP^26^ or COVID-19 severity^9^ in other studies, further supporting our findings. Owing to the contribution of bacterial co-infections to mortality and heightened disease severity in COVID-19 infections, oropharyngeal bacteria are expected to be used as robust predictors of COVID-19 severity and as intervention targets.

Moreover, the oropharyngeal microbiome of COVID-19 patients was characterized by significant changes in the degradation of amino acids and other small molecules. Amino acid imbalance was reported to increase intestinal inflammation via ACE2-dependent changes in epithelial immunity.^36^ Thus, imbalanced metabolites may cause changes in the immune microenvironment and increase the burden of COVID-19. In addition, we observed a dramatic increase in antibiotic resistance genes in the oropharyngeal microbiome of COVID-19 patients, especially in the critical patient group. Antibiotic resistance may slowly accumulate since most critical patients have multiple complex comorbidities and may have a history of high antibiotic intake. Antibiotic abuse may change the microbiome and slowly increase resistance, leading to bacterial resistance, which may be related to critical viral infection.

Dual and mutual interactions of the oropharyngeal microbiome with inflammation and the immune system during the onset of a disease are actively being investegated.^37^ Additional experiments are required to reveal the mechanistic effects or causative roles of the oropharyngeal microbiome and the changes in metagene functions in the susceptibility and severity of SARS-CoV-2 infection. However, unlike influenza virus which can be investigated in a germ-free mouse model, there are no appropriate animal models to mimic severe symptoms observed in COVID-19 patients, which makes functional investigations more difficult.

Evidence provided by us and other researchers indicates certain variations in the composition of the oropharyngeal microbiome caused by SARS-CoV-2 infection and suggests that indicator species within the oral ecosystem may be used as surrogate markers of the severity of COVID-19. The oral cavity is one of the first entry points in the body and a significant reservoir of SARS-CoV-2; hence, we can rationally infer that dysbiosis of the local airway microbiome induced by SARS-CoV-2 infection initially occurs in the oral cavity and subsequently impacts distant microbiomes across connected body sites via the oral–lung or oral–gut axis.^38^ Therefore, manipulation of the oropharyngeal microbiome may be a potential prevention strategy.

Thus, the results of the present study provide potentially significant clinical findings. First, the data reveal that the variation in the oropharyngeal microbiome in COVID-19 may be used as a noninvasive biomarker for dysbiosis of the pulmonary microbiome or for invasion of potential pathogens in the lung; second, we provide some evidence that major potential pathogens are associated with lung co-infections in COVID-19 to guide antibiotic treatment of secondary bacterial infections in COVID-19; third, bacterial strains, such as *Veillonella parvula*, identified in the present study require future studies to determine their roles in the pathogenicity of SARS-CoV-2 and in COVID-19 development; fourth, the data provided a compelling rationale suggesting that effective oral hygiene measures and promotions are necessary to reduce secondary infections, especially in patients with severe COVID-19.

## Materials and methods

### Overview of enrollment

The basic clinical information of the cohort (31 confirmed patients with COVID-19, 29 flu patients with influenza B, and 28 healthy controls) is shown in Supplementary Table 1. One COVID-19 patient and all the flu patients and healthy controls were admitted to Heilongjiang Provincial Hospital from Jan 20^th^ to Feb 25^th^, 2020, and 30 COVID-19 patients were admitted to Suihua First Hospital and Suihua Cancer Hospital from Jan 24^th^ to Feb 25^th^, 2020.

The patients were categorized into four groups based on disease severity, i.e., mild, moderate, severe, and critical cases, according to the Diagnosis and Treatment Protocol for Novel Coronavirus Pneumonia (Trial Version 7). Briefly, there was no sign of pneumonia on imaging in mild cases. Moderate cases were defined as showing fever and respiratory symptoms with radiological findings of pneumonia. Severe cases were defined by meeting one of the following criteria: respiratory distress ≥30 breaths/min, oxygen saturation ≤93% at rest, arterial partial pressure of oxygen (PaO_2_)/fraction of inspired oxygen (FiO_2_) ≤300 mmHg or chest imaging showing obvious lesion progression within 24–48 h >50%. Critical cases were defined as one of the following: respiratory failure requiring mechanical ventilation, shock, or other organ failure requiring intensive care unit admission.

### Sample collection and metagenomic sequencing

The COVID-19 and flu patients and healthy controls gargled with clean water, and mucosal cells were collected by a doctor after applying disposable sterile sampling cotton swabs to the posterior pharynx, sidewalls, and crypts of the tonsil and wiping three to five times in a rotating manner. Then, the cotton swab was placed into an oral swab preservation tube (purchased from Kangwei Century Biotechnology Co., Ltd.).

Microbial nucleic acids were extracted from 88 oropharynx swab samples by a TIANamp Micro DNA Kit (DP316, TIANGEN BIOTECH) according to the manufacturer’s recommendations after the host cells were removed with a self-developed host-removal kit. Then, DNA libraries were constructed with DNA fragmentation, end repair, adapter ligation, and PCR amplification. An Agilent 2100 was used for quality control of the DNA libraries. Quality-controlled libraries were sequenced with a MGISEQ-2000 platform.

### Bioinformatics analysis

The original sequencing data were processed as follows to obtain clean data. Reads containing 10% uncertain bases (*N* bases) were removed; reads containing sequencing adapter sequences (15 bases or longer regions mapped to the adaptor sequence) were removed; reads containing more than 50% low-quality bases (bases with Q < 20) were eliminated; and SOAP2^39^ alignment was used to remove reads that mapped to the human genome with more than 90% similarity.

The taxonomic classification of clean reads and calculation of taxonomic abundance were performed using Kraken2 v2.0.9^40^ with the provided prebuilt Maxikraken2 databases (last updated in March 2019). Differentiating taxa by pairwise comparisons or the comparison of three groups was performed with the Wilcoxon rank sum test^41^ and kw test,^42^ respectively. Correlations between taxa and clinical characteristics were tested using Spearman correlation analysis, and visualization of the network was performed by Cytoscape. To further analyze species that contribute to the discrimination of different group samples, a random forest analysis, as implemented in QIIME 2, was used. The ROC curves were plotted for the classification performance of the trained random forest models with sklearn at the genus and species levels. Spearman correlation analysis between different genera was performed with the R package “Hmisc.” All plots and statistical analyses were conducted with R v4.0.0, and the vegan package in R was utilized to obtain the diversity indexes, including the Shannon index for alpha diversity and Bray–Curtis dissimilarities for beta diversity.

MEGAHIT^43^ was used to perform the de novo assembly of each sample, and contigs larger than 150 bp were retained for gene prediction by MetaGeneMark3^44^ and CD-Hit4.^45^ Briefly, MetaGeneMark3 (version 2.10, default parameters) was used to predict the open reading frame, and then CD-Hit4 was applied for gene clustering and merging each sample. Finally, redundant sequences with sequence similarity above 95% and alignment lengths >90% of the sequence length were removed.

Resistance Gene Identifier^46^ (version number: 3.2.1) was used to identify AMR genes. Differentially enriched KEGG ortholog (KO) pathways were identified based on their reporter score from the *Z*-scores of individual KOs (KEGG database release 89.1). The reporter score was calculated as follows: the *P* value of the KO was obtained by the rank sum test, and the *Z* value corresponding to the *P* value was obtained using an inverse normal distribution.^47^

## Supplementary information

Supplementary TableS6

supplementary materials

Supplementary TableS1

Supplementary TableS2

Supplementary TableS3

Supplementary TableS4

Supplementary TableS5

## Data Availability

The metagenomics sequencing dataset was deposited in the China National GeneBank Nucleotide Sequence Archive BioProject accession number CNP0001259.
