# Supplementary material for: Metagenomic analysis reveals oropharyngeal microbiota alterations in patients with COVID-19
Source: Signal Transduct Target Ther. 2021 May 13;6:191. doi: 10.1038/s41392-021-00614-3 (PMC8116522; doi:10.1038/s41392-021-00614-3)
Supplement: Supplementary file 2 — supplementary materials [file 41392_2021_614_MOESM2_ESM.docx]

Supplementary Materials for

Metagenomic analysis reveals throat microbiota alterations in patients with COVID-19

Shengli Ma^1^*, Fan Zhang^2^, Fengxia Zhou^2^, Hui Li^1^, Wenyu Ge^1^, Rui Gan^2^, Huan Nie^2^, Biao Li^3^, Yindong Wang^1^, Meng Wu^1^, Duo Li^4^, Dongmei Wang^1^, Zheng Wang^1^, and Yuhong You^1^, and Zhiwei Huang^2^*

Correspondence: Shengli Ma (msl6377@aliyun.com) or Zhiwei Huang (huangzhiwei@hit.edu.cn)

**This PDF file includes:**

Figures. S1

Other supplementary materials for this manuscript include the following:

**Table S1.** Clinical features of enrolled patients and healthy subgroups.

**Table S2.** Clean reads for metagenome assemble and species annotation.

**Table S3.** The statistical result of metagenome assembly and gene annotation.

**Table S4.** Differentially abundance species in the three groups.

**Table S5.** Correlation between clinical characteristics and microbiota in COVID-19 patients at species level.

**Table S6.** KEGG pathway enrichment of 7,842,539 proteins in landscape oral human microbiome.


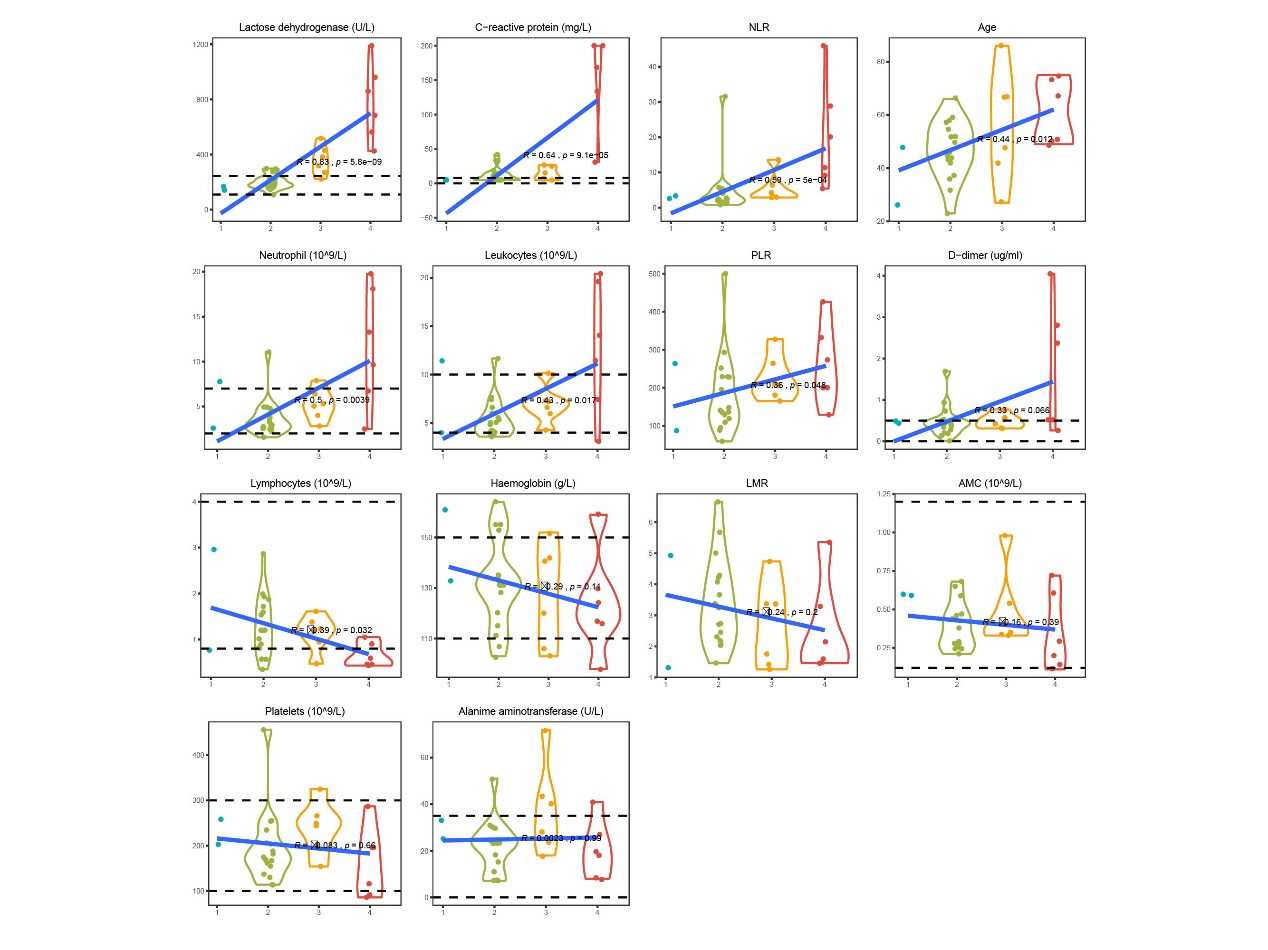


Figure. S1.

Violin plot showing the correlation between clinical characteristics in Supplementary Table 1 and the severity of COVID-19 patients, the spearman correlation is used for correlation analysis, dashed horizontal lines reflect the reference ranges for those clinical characteristics.
